# The Rheology behind Stress-Induced Solidification in Native Silk Feedstocks

**DOI:** 10.3390/ijms17111812

**Published:** 2016-10-29

**Authors:** Peter R. Laity, Chris Holland

**Affiliations:** Department of Materials Science and Engineering, The University of Sheffield, Sir Robert Hadfield Building, Mappin Street, Sheffield S1 3JD, UK

**Keywords:** native silk feedstock, rheology, flow-induced phase change, thermally-induced phase change

## Abstract

The mechanism by which native silk feedstocks are converted to solid fibres in nature has attracted much interest. To address this question, the present work used rheology to investigate the gelation of *Bombyx mori* native silk feedstock. Exceeding a critical shear stress appeared to be more important than shear rate, during flow-induced initiation. Compositional changes (salts, pH etc.,) were not required, although their possible role in vivo is not excluded. Moreover, after successful initiation, gel strength continued to increase over a considerable time under effectively quiescent conditions, without requiring further application of the initial stimulus. Gelation by elevated temperature or freezing was also observed. Prior to gelation, literature suggests that silk protein adopts a random coil configuration, which argued against the conventional explanation of gelation, based on hydrophilic and hydrophobic interactions. Instead, a new hypothesis is presented, based on entropically-driven loss of hydration, which appears to explain the apparently diverse methods by which silk feedstocks can be gelled.

## 1. Introduction

Silk has been a remarkable source of wonder throughout human history. This family of natural protein fibres, which is widely produced by all spiders [1,2,3,4], many insects [2,5,6,7,8,9,10] and some other arthropods [2,11,12,13], has provided inspiration for artists [14], resources for traders [15,16,17] and motivation for scientists [18,19,20,21], from its earliest use for textiles in China [17], to the latest scientific research into advanced biomaterials and other uses [22,23,24,25,26,27]. Much of this endeavour has concentrated on the impressive mechanical properties of fibres produced by Lepidopteran larvae (i.e., the domesticated *Bombyx mori* (*B. mori*) and other wild silk moths) or spiders [4,21,28,29,30,31,32,33,34,35]. Whilst acknowledging the importance of that work, however, this paper proposes the solidification mechanism, by which silk fibres are produced, to be the most fascinating aspect.

In contrast to other protein fibres (e.g., hair or wool) grown by animals, silk is produced on demand, by spinning from an aqueous feedstock solution. Although this feedstock seems to be effectively stable within the animal, the (liquid to solid) phase change appears to be initiated merely by flow through the spinning apparatus [18,19,20,36,37,38,39,40,41,42,43,44]. Thus, silk is produced under ambient conditions, with minimal energy consumption, in a water-based system, without the involvement of hazardous or exotic chemicals. By contrast, conventional man-made fibres generally require high temperatures (for melt- or dry-spinning), solvents or chemical derivatisation (for wet-spinning) [45,46,47], with corresponding energy costs for process heating, production of ancillary chemicals, solvent recovery (where appropriate) and environmental protection.

An explanation for flow-induced coagulation was proposed by Iizuka [40,41,42], in which stresses during spinning unfolded the silk protein molecules, exposing charged groups, promoting aggregation and leading to β-sheet formation. An alternative explanation was offered by Jin and Kaplan [43], where the *B. mori* fibroin was described as a copolymer with alternating hydrophilic and hydrophobic segments, based on the hydropathy index concept of Kyte and Doolitle [48]; flow stress caused the protein to unfold and initiate gelation through hydrophobic interactions. These explanations are not entirely satisfactory, however, as a well-defined tertiary structure appears to be inconsistent with the predominantly disordered or random coil configuration of native fibroin indicated by circular dichroism (CD) [49,50,51,52,53] and nuclear magnetic resonance (NMR) [54,55,56]. In particular, NMR indicated a strong preference for β-turns and fibroin chains exhibiting fast segmental motion typical of a random coil polymer, even in a highly entangled state at the concentration of native silk feedstock within the silkworm. In this respect, the native state of fibroin may have similarities to the intrinsically disordered proteins (IDPs), which have gained attention in recent years [57,58].

Nevertheless, the importance of a flow-induced phase change at a critical shear rate or stress has motivated a considerable number of rheological investigations [36,37,59,60,61,62,63,64,65,66,67,68,69,70,71,72]. Amongst these, recent work has demonstrated that *B. mori* silk feedstock behaves as a typical polymer solution [59,60,61]. Its flow behaviour was found to be dominated by a relatively small number of relaxation modes and followed an Arrhenius-type temperature dependence [59]. Moreover, although considerable sample-to-sample variations have been observed, normalisation with respect to the cross-over frequency (ω_X_) and modulus (*G*_X_) appeared to reduce the oscillatory data onto a master-curve [60].

The phase change of silk feedstock can also be initiated in various other ways. Heating causes solidification [70,73,74], with apparent similarities to cooking an egg. This example of molecular gastronomy is usually explained in terms of heat causing an unfolding (“melting”) of the native protein structure (denaturing): hydrophobic amino-acids that were originally shielded become exposed and an extended intermolecular network forms through the resulting interactions [75,76,77]. The origin of this classical proposal can be found in a review paper by Mirsky and Pauling [78]. Although it may provide a plausible explanation for globular proteins with hydrophilic and hydrophobic regions within well-defined tertiary structures in their native states, it appears inappropriate for *B. mori* fibroin, which appears to exhibit a random coil structure, as already discussed. Instead, the thermal gelation of fibroin may be more related to the gradual decrease in coil dimensions shown by IDPs during heating, which contrasts with the sudden expansion over a narrow temperature range shown by “conventional” proteins [58,79]. Although less well studied, silk feedstocks can also be gelled through freezing [80], which occurs presumably without any melting of the tertiary structure. Again, in apparently unrelated ways, gelation can be induced through the actions of salts, acids or water-miscible non-solvents [81,82]. Nevertheless, the precise details of these phase change mechanisms are still subject to much conjecture.

The majority of the work presented here explored the flow-induced phase change of native silk feedstock specimens from *B. mori*. Combinations of shear- and oscillatory rheology were used to investigate the existence of a critical shear rate or stress and the subsequent train of events during gelation. In particular, our results indicated shear stress to be a more consistent indicator than shear rate. For comparison, the phase changes initiated by heating or freezing were also studied. In order to explain these observations, without recourse to the unsatisfactory assumption of hydrophilic and hydrophobic regions in the protein, a new hypothesis is presented; this is based on native fibroin being stabilised by a hydration shell, which can be displaced as a consequence of entropy changes, initiating coagulation. Moreover, it is suggested that this explanation can also unite the otherwise disparate observations of temperature-, salt-, acid- or non-solvent-induced gelation.

## 2. Results

**Figure 1:**
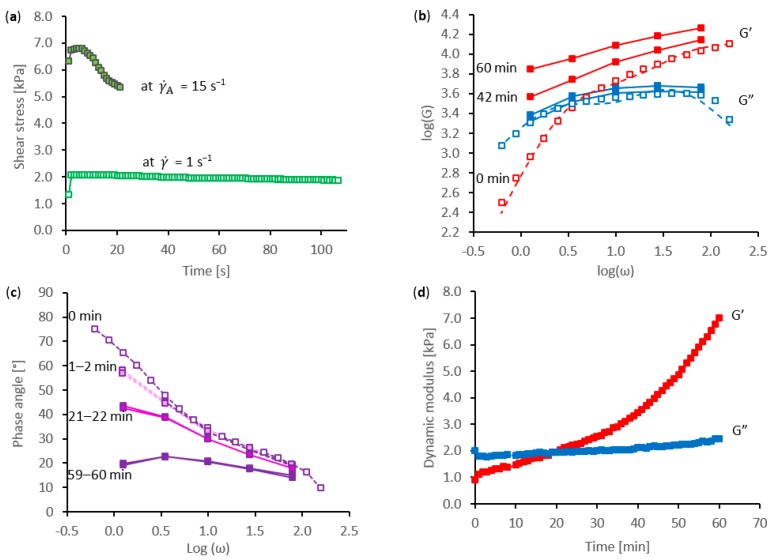
Rheological demonstration of shear-induced phase change: (**a**) shear stress during steady flow at shear rates of 1 and 15 s^−1^, where the latter period of high shear stress initiated the phase change; (**b**) examples of dynamic moduli; (**c**) examples of frequency dependence of the phase angle; (**d**) progressive changes in dynamic moduli measured at 0.2 Hz (1.25 rad·s^−1^). Open points indicate initial data prior to initiation; filled points indicate data during the phase change.

The rheological changes associated with flow-induced gelation of native silk feedstock were observed by a combination of shear and oscillatory measurements, during experiments performed in several stages. Following characterisation by constant shear flow (100 s at a shear rate of $\dot{\gamma }$ = 1 s^−1^) and an oscillatory sweep (17 points covering the frequency range *f* = 25 to 0.1 Hz, over 5 min), the specimen was subjected to a short period of faster shear flow and the resulting changes were observed by repeated shorter oscillatory sweeps (5 points from 12.5 to 0.2 Hz, in less than 1 min). Typical results are presented in Figure 1.

For the material in its initial state, viscosity measurements during steady flow at a shear rate ($\dot{\gamma }$) of 1 s^−1^ showed a shallow peak in the shear stress (σ) approximately 5 s after the start of flow, followed by a gradual decrease at a roughly constant rate over the rest of the measurement period, as shown in Figure 1a. This was similar to the behavior reported previously [59,60,61]. The initial peak was ascribed to “stress overshoot”, which is a non-linear rheological effect commonly observed with polymeric systems and generally ascribed to the changes in coil shape at the onset of steady flow [83,84,85,86,87,88]. The subsequent prolonged decrease over the duration of the measurements may indicate other causes, however, such as the gradual dilution or dissolution of excess material into the water surrounding the sample area or a re-distribution of the specimen between the cone and plate due to normal stress differences. In spite of this longer-term downward trend, the individual shear stress measurements appeared quite consistent, indicating stable flow behavior.

During the final 30 s at $\dot{\gamma }$ = 1 s^−1^, an average shear stress of 1.89 kPa was observed, corresponding to a viscosity (η_1_ = σ/$\dot{\gamma }$) of 1890 Pa·s. Although previous work demonstrated considerable natural variation between specimens of silk feedstock from *B. mori*, this value was close to the median value expected during the early stages of cocoon construction [61].

The dynamic moduli (measured in oscillation) are shown in Figure 1b. Following the initial shear flow at 1 s^−1^, the oscillatory data (shown by open points in Figure 1b) revealed viscoelastic behaviour, typical of a polymer solution. The elastic modulus (*G*’) dominated at higher frequencies, while the viscous modulus (*G*”) dominated at lower frequencies. As reported previously [59,60,61], the dynamic modulus data could be fitted well using a Maxwellian model of conceptual springs and dash-pots based on only two relaxation modes:

(1a) $$G^{\prime }=\sum_{i=3}^{4}g_{i}\frac{\omega^{2}\tau_{i}^{2}}{1+\omega^{2}\tau_{i}^{2}}$$

(1b) $$G^{\prime \prime }=\sum_{i=3}^{4}g_{i}\frac{\omega \tau_{i}}{1+\omega^{2}\tau_{i}^{2}}$$

where ω is the angular frequency (equal to 2π*f*), *τ_i_* is the relaxation time and *g_i_* represents the contribution of that mode to the dynamic modulus. These modes were designated 3 and 4, in line with previous reports [59,60,61], as two slower modes (designated 1 and 2) were revealed by quasi-static stress relaxation measurements. This relatively simple model is represented by the dashed lines in Figure 1b.

Considerable changes were observed during the subsequent shear flow measurements (over 20 s at 15 s^−1^, in this example). The shear stress achieved a peak of 6.77 kPa at around 7 s, then fell to 5.34 kPa by the end of the measurements, corresponding to an apparent decrease in the viscosity at this shear rate (η_15_) from 451 to 356 Pa·s. Although these changes in shear stress were considerably larger and faster than those observed at 1 s^−1^, they may have originated from similar effects: stress overshoot and changes in sample distribution in the geometry are often observed to be more pronounced at higher shear-rates [83,84,85,86,87]. On the other hand, errors due to “wall slip” or “edge fracture” [89] could be more significant at the higher shear rate.

Moreover, while the shear stress increased with shear rate, it corresponded to a decrease in viscosity, in line with previous reports on silk feedstocks by other workers [36,40,42,62,63,64,65,66,67,68,69,70]. Shear thinning is commonly observed for polymer solutions or melts, although a definitive explanation is still somewhat elusive. This phenomenon is predicted by the “tube model”, through flow-induced changes in coil shape, “topological interactions” (i.e., entanglements) and “convective constraint release” [83,84,87,88,89,90,91]. For proteins, however, additional mechanisms based on the destruction of physical (e.g., ionic or dipolar) interactions between specific amino-acids cannot be ruled out.

For the example shown in Figure 1, the brief period of higher flow rate initiated a phase change, which was revealed in the subsequent oscillatory measurements. Over the duration of this part of the experiment (1 h) the plots of viscous modulus against frequency rose slightly, while the elastic modulus increased dramatically. This is shown (by filled points and continuous lines) in Figure 1b. Consequently, the cross-over between predominantly viscous (*G*” > *G*’) and elastic (*G*’ > *G*”) behaviour moved to progressively lower frequencies. Eventually, the cross-over fell beyond the low frequency limit of the measurements (0.2 Hz, for these shorter scans), consistent with a gradual phase change and gel formation.

A clearer representation of these changes is given by the frequency dependence of the phase angle (δ), which describes the phase lag between the viscous and elastic components of the complex modulus, where:

(2) $$\text{tan }\delta =\frac{G\prime \prime }{G\prime }$$

Note that values of δ above 45° indicate tanδ > 1 and *G*” > *G*’ (predominantly viscous behaviour) while values of *δ* below 45° indicate *G*’ > *G*” (predominantly elastic behaviour).

There appeared to be an abrupt decrease in *δ* at lower frequencies, between the data collected prior to and immediately following the higher shear rate flow (labelled 0 and 1 to 2 min in Figure 1c). This was linked to a slight reduction in *G*” together with an increase in *G*’ and suggested an immediate change in the relaxation behaviour as a result of the previous constant shear flow. Much smaller changes were observed at higher frequencies, suggesting that the slower relaxation processes were mainly affected. This is explored further in Section 2.3.

In spite of the rather limited data collected during the rapid frequency scans and the changing rheology during the ensuing gelation, the individual measurements appeared reliable. Hence, there was good agreement between consecutive scans, as demonstrated by comparing the plots of phase angle against frequency from 1 to 2 min, 21 to 22 min or 59 to 60 min, which are shown in Figure 1c.

The phase angle (measured at 0.2 Hz) decreased progressively over this part of the experiment, due to the elastic modulus increasing faster than the viscous modulus (as shown in Figure 1d). Nevertheless, the values of δ remained above 45° for a considerable period (roughly 20 min in this example), indicating that the specimen was still in a liquid state immediately following the higher shear rate flow. Predominantly elastic behaviour (δ < 45°, indicating *G*’ > *G*”) became evident beyond 20 min and continued to develop throughout the rest of the experiment. Ultimately, when the apparatus was cleaned, it was found that the initially clear, colourless liquid specimen had become turbid and solid, which confirmed the rheological indications of phase change.

Several key inferences can be drawn from these results. Firstly, it confirms previous observations that the phase change and the gelation of silk feedstock can be initiated simply by shear flow [36,37,38,39,40,41,42,43,44]; changes in ionic content, protein concentration, pH, the presence of sericin or other chemical constituents were not required in these experiments, although that does not preclude their possible involvement in natural silk spinning. Secondly, once initiated, the phase change can take place under essentially quiescent conditions without needing further external stimulation. Moreover, the gelation may not be instantaneous, but can evolve progressively over a considerable time-period (beyond 60 min in the example given). In this respect, it may be noted that Li et al. [52] proposed a nucleation and growth mechanism, while Matsumoto et al. [49] suggested a two-stage route of weak interactions followed by β-sheet formation for gel formation in (regenerated) silk fibroin solutions. A two-stage process of protein unfolding followed by aggregation also fits with the classical model of protein gelation [75,76].

### 2.1. Critical Flow Conditions

In the foregoing example, the combination of viscosity and shear rate initiated a phase change, resulting in the gradual gelation and eventual solidification of that specimen. By observing the outcomes from different viscosity and shear rate combinations, it was possible to explore the conditions required to initiate such phase change. An important constraint was that the viscosity of each specimen was controlled by the silkworm. As reported previously [61], this could vary considerably and the viscosity of an individual specimen was not known in advance of starting the experiment. Consequently, in order to control the conditions explored, the experiments were performed in two stages: first, the specimen was characterized by the usual shear flow and oscillatory measurements; then appropriate higher flow conditions were selected for that specimen, followed by more oscillatory measurements to observe whether a phase change ensued.

**Figure 2:**
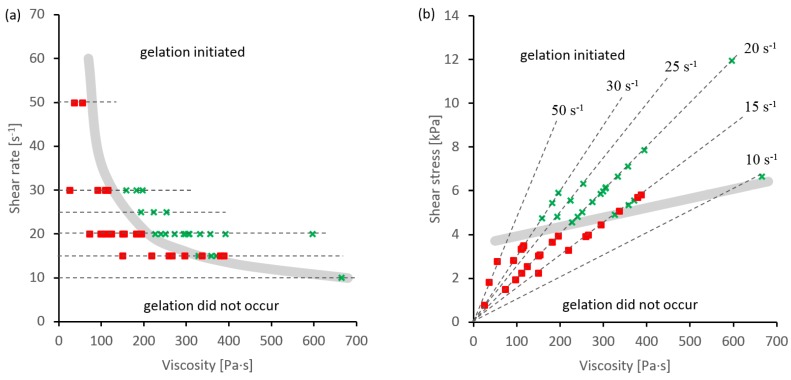
Maps showing flow conditions that did (green crosses) or did not (red filled squares) initiate phase changes, plotted with respect to: (**a**) viscosity and shear rate; (**b**) viscosity and shear stress. Thick grey lines indicate (approximate) boundaries between successful and unsuccessful initiation; dashed lines indicate experiments using the same shear rates. The viscosities were measured at the shear rates indicated.

Hence, it was possible to map the effects of shear flow on subsequent phase change behavior, as shown in Figure 2. In both graphs, the horizontal axes represent the shear viscosity under the higher flow rate conditions, while the vertical axes represent shear rate (Figure 2a) or shear stress (Figure 2b). Due to the relationship between the viscosity, shear rate and shear stress, the results fell along horizontal or sloping lines (in Figure 2a,b respectively). It should also be noted that the shear rate (${\dot{\gamma }}_{A}$) and duration (*t*_A_) of the initiation period were selected to maintain a constant value of total shear (i.e., $t_{A}.{\dot{\gamma }}_{A}$ = 300 in all these experiments).

All combinations involving high viscosity and high shear rate or stress produced significant changes in rheology, similar to those demonstrated in Figure 1. In particular, the phase angle at low frequency decreased, while the elastic modulus increased, indicating that phase change had been initiated. Conversely, combinations involving low viscosity and low shear rate or stress did not initiate a phase change. In those cases, the phase angle and elastic modulus remained close to the values observed initially in the specimen. These “failed” experiments actually demonstrate several important points. Firstly, the initial periods of low shear rate flow and oscillatory measurements were common to all these experiments; hence, they were not a deciding factor in the eventual outcome. Secondly, all experiments were conducted over similar time-scales, which argues against the phase change being merely the result of the specimen remaining on the rheometer for a sufficient duration. Finally, all oscillatory measurements—including those used to monitor the outcome—were performed with a strain amplitude of 0.02, which represented effectively zero-flow conditions during the phase change and was set to be within the material’s linear viscoelastic region [65].

By conducting a sufficient number of experiments with appropriate flow conditions, it was possible to locate the boundary between the *status quo ante* and incipient phase change; this is plotted as the thick grey lines in Figure 2a,b. Considering the shear rate (Figure 2a), the boundary decreased along a curved line, as the viscosity increased. A shear rate above 30 s^−1^ was required to initiate a phase change in a specimen with a viscosity of 100 Pa·s, whereas 10 s^−1^ was sufficient for a specimen with a viscosity of 700 Pa·s. This contradicts the existence of a definite and fixed critical shear rate; instead, it is clear that initiation depended on both the viscosity of the specimen and the shear rate used.

A more consistent picture emerged by considering the shear stress (Figure 2b). In this case, the boundary increased slightly with viscosity, from around 4.0 kPa at 100 Pa·s to 6.5 kPa at 600 Pa·s. Thus, although the shear rate appeared to play some part, exceeding a critical shear stress was a more important criterion for initiating the phase change. Closer examination of the results also revealed some uncertainty under “borderline conditions” close to the estimated boundary. This may have been due to other “experimental” factors such as stresses during sample preparation and loading, or “physiological” changes in the silk feedstock.

### 2.2. Effect of Initiation Conditions on the Rate of Gelation

**Figure 3:**
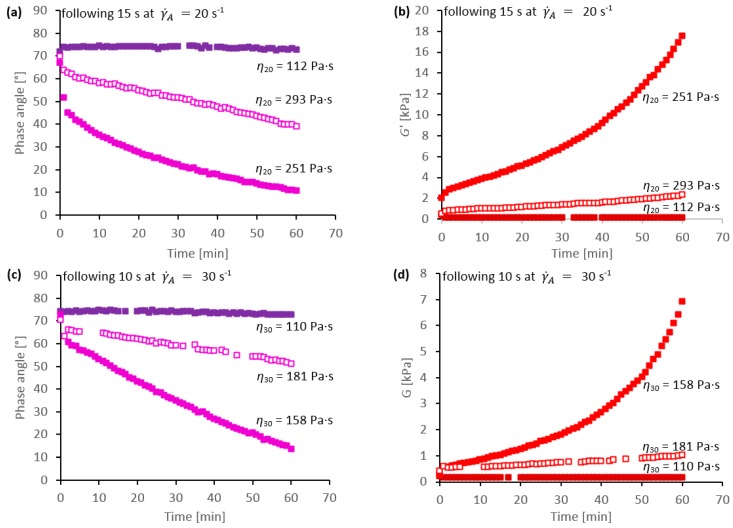
Examples of rheological behaviour for different feedstock specimens, following (successful or unsuccessful) attempts at flow-initiated phase changes: plots of (**a**) phase angle and (**b**) elastic modulus at 0.2 Hz vs. time, following 15 s period of flow at a shear rate of 20 s^−1^; (**c**,**d**) corresponding data following 10 s flow at 30 s^−1^. The viscosities quoted for each specimen were determined at the end of the period of initiation flow, at the shear rate shown.

The results shown in Figure 2 demonstrate the conditions required to initiate the solid-to-liquid phase change, but pay little attention to the rate at which the subsequent gelation occurred. This is explored in Figure 3, in terms of the changes in phase angle and elastic modulus over time. Considerable differences in the rates of change of gelation were found between specimens, with some forming gels (i.e., achieving *G*” < *G*’, even at low frequency) within a few minutes, while others took an hour or more.

It was anticipated that the rates of change (in terms of the values of δ or *G*’) might be greater if the gelation were initiated further from the boundaries and deeper into the effective areas shown in Figure 2. Hence, for any given shear rate, specimens with higher viscosities would be expected to exhibit faster changes. That was not found, however. Of the examples shown in Figure 3, it appeared that the specimen with η_20_ = 251 Pa·s changed faster than that with η_20_ = 293 Pa·s (Figure 3a,b), while the specimen with η_30_ = 158 Pa·s changed faster than that with η_30_ = 181 Pa·s (Figure 3c,d). Moreover, similar apparent inconsistencies were also found by examining a larger set of results.

One possible explanation is that the viscosity measurements at higher shear rates were unreliable. Clearly, from the example shown in Figure 1a (at ${\dot{\gamma }}_{A}$ = 15 s^−1^), the viscosity exhibited significant changes during the shear flow. Although non-linear rheological effects of this kind are commonly observed with polymeric systems, ascribed to changes in coil geometry during the onset of shear flow, it brings into question the meaning of an instantaneous value for the viscosity. Also, measurements at higher shear rates may have incurred greater errors due to “wall slip” or edge fracture [89].

Secondly, it is worth reiterating that the initiation of phase change by suitable flow conditions and the subsequent evolution of the gel appeared to occur sequentially, as two separate processes. Hence, the best conditions for initiation (high shear rate and viscosity, producing high shear stress) may not be optimal for the subsequent gel formation. In this respect, it may be useful to consider the possible changes taking place within the silk feedstock. For example, the onset of gelation may have involved the formation of an interconnected network of protein chains, which could have been disrupted by additional flow. Hence, this would imply the existence of “optimal conditions” for initiating phase change, requiring just enough, but not excessive flow. It is also assumed that the flow behaviour within the specimen was homogeneous, such that the rheological data reflected the entire specimen. On the contrary, it is possible that the phase change may have started locally, within small regions of the specimen, in line with the “shear dependent silk fibrillogenesis” reported by Holland et al. [36]. Hence, the rates of subsequent changes in phase angle or elastic modulus might reflect how well the gelled structures percolated through the specimen.

### 2.3. The Role of Slow Relaxation Modes

**Figure 4:**
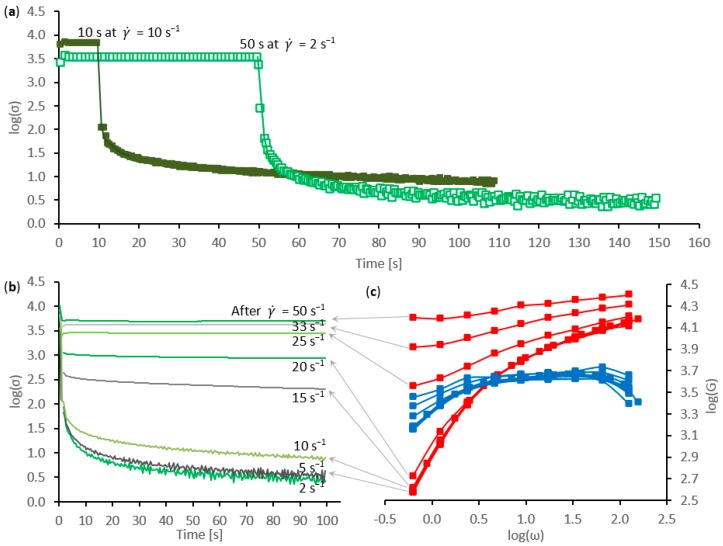
Sequences of stress and oscillatory measurements for a silk feedstock specimen during shear-induced phase change: (**a**) shear stress during constant shear flow (at $\dot{\gamma }$ = 2 or 10 s^−1^, corresponding to a total strain of 100), followed by quasi-static relaxation (over 100 s at $\dot{\gamma }$ = 0.0005 s^−1^); (**b**) comparison between quasi-static stress relaxation and (**c**) oscillatory data (*G*’ in red, *G*” in blue), for a silk feedstock sample following periods of shear flow at increasing shear rates.

Previous work [61] revealed the existence of slow relaxation modes that could be evaluated from stress decay measurements following the cessation of shear flow. Although it was not possible to perform those measurements under completely static conditions, with the apparatus used, the stress decay could be followed under quasi-static conditions, at $\dot{\gamma }$ = 0.0005 s^−1^. Typical results are shown in Figure 4.

During the periods of faster shear flow (at 2 and 10 s^−1^, for the example shown in Figure 4a), the stress increased with shear rate, in line with expectations. Although these sections of the graphs (using a logarithmic stress axis) appeared fairly flat, the data exhibited the same time-dependent changes as shown in Figure 1a. Then, following the cessation of faster flow (i.e., a sudden drop to $\dot{\gamma }$ = 0.0005 s^−1^) the stress decayed exponentially with time, following expressions of the type:

(3) σ=∑i=14σi.exp(−tτi)

where *σ_i_* and *τ_i_* are stress contributions and relaxation time constants. The values for the faster modes (3 and 4) were estimated from oscillatory measurements performed immediately after the quasi-static period; thus, the parameters describing the slower modes (1 and 2) could be evaluated by fitting this model to stress relaxation data.

Initially, following periods of relatively slow shear flow ($\dot{\gamma }$ < 5 s^−1^), the stress decayed away almost completely (over 100 s at $\dot{\gamma }$ = 0.0005 s^−1^), as shown in Figure 4b. Subsequent oscillatory sweeps (in Figure 4c) indicated that flow behavior was essentially unchanged from the starting material. Following periods of faster shear flow, however, the slower modes became more prominent, with increases in both stress contributions and relaxation time constants. Ultimately, this resulted in a significant residual stress that did not decay during the quasi-static relaxation period. Interestingly, similar observations were also reported by Iizuka [42], although they seem to have received little attention in the intervening 50 years.

This increase in residual stress was accompanied by changes in the dynamic moduli, as shown in Figure 4c. While *G*” increased slightly, *G*’ increased dramatically, particularly at low frequency, corresponding to the onset of gelation. It may be noted, however, that the residual stress appeared to be considerably more sensitive to incipient phase change than the oscillatory data. For the example shown, a significant increase in the former occurred (corresponding to 7.3 Pa) following shear flow at 10 s^−1^, while changes in the dynamic moduli could only be observed after flow at 20 s^−1^. This may be ascribed to the stress relaxation observing much lower frequencies (<0.01 Hz) than accessible in the oscillatory measurements.

**Figure 5:**
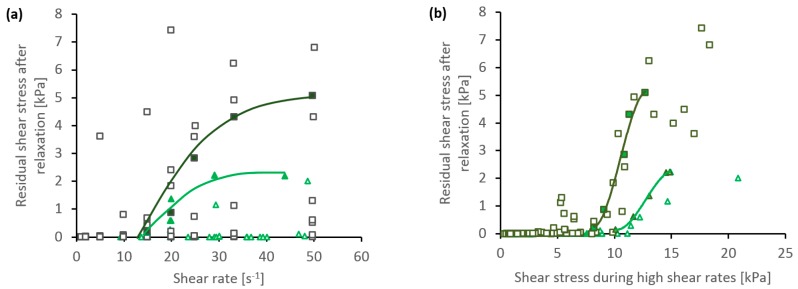
Comparison between residual stress after quasi-static relaxation (100 s at $\dot{\gamma }$ = 0.0005 s^−1^) and (**a**) shear rate or (**b**) shear stress during the periods of faster flow (of total strain 30 or 100, shown as triangles or squares, respectively). Filled points represent exemplar data from individual experiments; open points represent aggregated data from several experiments. The continuous lines show trend lines for the exemplars.

The effects of faster shear flow on the subsequent relaxation are also demonstrated in Figure 5. Attempts to plot the data against shear rate produced a rather confusing distribution of points (in Figure 5a). Data from individual experiments showed that the residual stress generally increased with the shear rate used, as demonstrated by the exemplars (shown by filled points and trend lines in Figure 5a). This was also consistent with the results shown in Figure 4b. On the other hand, comparisons between experiments (17 in total) revealed considerable discrepancies: some specimens retained large levels of residual stress after relatively slow flow, while others still relaxed completely after relatively fast flows. This may be ascribed to differences in viscosities between the specimens used (ranging from 320 to 8124 Pa·s at $\dot{\gamma }$ = 1 s^−1^), which affected the stress achieved at a given shear rate.

Hence, a clearer picture emerged by plotting the data against shear stress (in Figure 5b). In this case, it was found that flows producing shear stress below 5 kPa were followed by essentially complete relaxation, while a shear stress in excess of 12 kPa always produced residual stress. Some variation was observed between different specimens, giving rise to a modest spread of data in Figure 5b—albeit, considerably less than that in Figure 5a. As before, individual experiments showed that faster flows producing higher shear stress resulted in larger values of residual stress; this is demonstrated by the exemplars (filled points and trend lines, in Figure 5). Moreover, experiments using a total strain of 100 (shown as squares) appeared to consistently produce a stronger response than with a total strain of 30 (shown as triangles). Thus, it appears that both the rate of doing work (related to flow stress) and the amount of work may influence the phase change, in line with previous observations [20].

### 2.4. Phase Change at High and Low Temperatures

**Figure 6:**
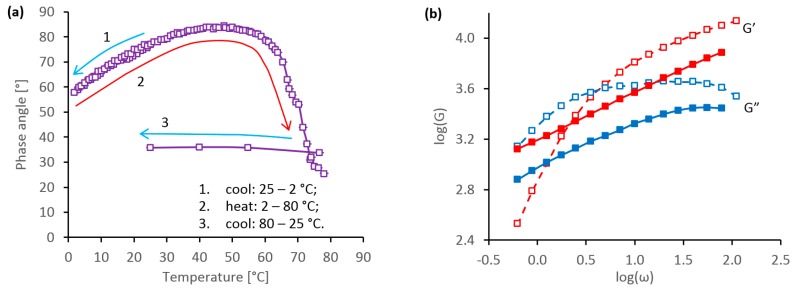
Rheological behaviour during thermal gelation of a silk feedstock specimen at elevated temperature: (**a**) changes in phase angle at 0.1 Hz, during cooling from 25 to 2 °C, subsequent heating from 2 to 80 °C and, finally, cooling from 80 to 25 °C; (**b**) comparison between oscillatory data at 25 °C, prior to (open symbols and dashed lines) and after (filled symbols and solid lines) the temperature cycle shown in (**a**). The arrows in (**a**) indicate the direction of temperature change.

The effect of heat on a typical specimen of silk feedstock is demonstrated in Figure 6. The specimen was loaded onto the rheometer at 25 °C, then cooled to 2 °C, heated to 80 °C and, finally, cooled back to 25 °C. The changes in rheology during this temperature cycle are demonstrated by the phase angle (measured at 0.1 Hz) in Figure 6a. The changes below 25 °C appeared to be essentially reversible, corresponding to the specimen becoming less fluid at lower temperatures. This trend appeared to continue to around 50 °C, with further increases in δ corresponding to the specimen becoming more fluid as it was heated.

The onset of phase change was indicated by a progressive decrease in δ above 50 °C, which became more rapid above 60 °C. During this stage, the phase angle fell below 45°, indicating a transition to predominantly elastic behavior for temperatures above 65 °C. After reaching the peak temperature of 80 °C, cooling produced relatively little further change in δ, which remained below 35°. Hence, this indicated that once the phase change had occurred, the specimen remained solid-like, irrespective of further temperature changes.

This phase change was confirmed by the plots of dynamic moduli in Figure 6b, which were measured immediately before and after the temperature cycle. The initial data (open symbols and dashed line) showed considerable frequency dependence, with a cross-over between predominantly viscous behavior at lower frequencies and elastic behavior at higher frequencies. This was consistent with the viscoelastic behavior generally observed for silk feedstocks in their native state. Conversely, after the temperature cycle, the dynamic moduli (filled symbols and solid lines) showed less frequency dependence, with *G*’ > *G*” throughout, indicating more solid-like behavior following gelation.

Rather surprisingly, the absolute values of dynamic moduli measured before and after the temperature cycle appeared to be comparable, in spite of clear indications of phase change to a more gel-like material. This may have been due to a change in the effective sample volume, through differential thermal expansion or syneresis after gelation. It was generally observed that the phase change was accompanied by a large positive increase in the axial force (i.e., pushing the cone away from the plate), which became strongly negative (i.e., pulling the cone towards the plate) after returning to 25 °C. This may have caused a decrease in the strength of contact between the specimen and the tooling, allowing some slippage to occur.

Slight differences in the onset temperature for the phase change were observed between specimens. Nevertheless, the steepest change in δ always occurred between 55 and 65 °C, which agrees well with previous observations of a thermally induced phase change in *B. mori* silk feedstocks using differential scanning calorimetry [73,74].

**Figure 7:**
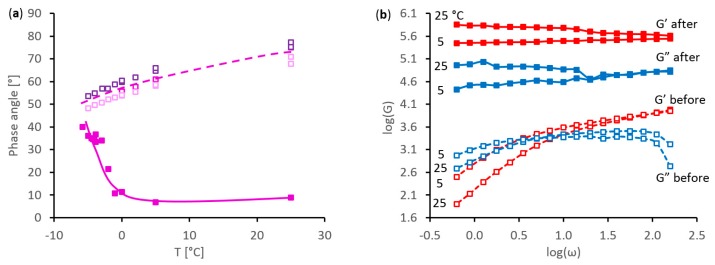
Rheological behaviour during gelation of a silk feedstock specimen due to freezing: (**a**) changes in phase angle (at 0.1 Hz) during cooling from 25 to −6 °C and subsequent heating from −6 to 25 °C; (**b**) comparison between oscillatory data measured at 25 and 5 °C, prior to (open symbols and dashed lines) and after (filled symbols and solid lines) the freezing cycle shown in (**a**). Different colours in (**a**) indicate different specimens; the open points show data prior to freezing, the filled points show data after freezing.

The effects of freezing are demonstrated in Figure 7. In this case, the specimen was loaded onto the rheometer at 25 °C, cooled then re-heated to 25 °C. Rheological changes during this process were characterised by periodic oscillatory sweeps at set temperatures. Feedstock specimens became more viscous as the temperature was lowered, yet they remained liquid (with δ > 45° at 0.1 Hz) down to −5 °C, as demonstrated by the open points and dashed line in Figure 7a. If the specimen was re-heated before it froze, the phase angle changed reversibly with temperature, indicating that gelation had not occurred.

Freezing was observed around −6 °C and was accompanied by a significant decrease in δ, as shown by the filled points and solid line in Figure 7a. It is not clear to what extent this sudden change in phase angle was due to the gelation of the silk feedstock, or ice formation within and around the specimen. Also, at the lowest temperature, control became somewhat unreliable, which caused some bunching of data for the frozen sample between −6 and −3 °C. On re-heating above 0 °C, however, the phase angle remained around 10°, indicating that gelation had occurred.

These changes were confirmed by the oscillatory data shown in Figure 7b. Prior to freezing, the dynamic moduli at 25 or 5 °C were characteristic of a viscoelastic material. For both *G*’ and *G*”, the data obtained at 5 °C lay above that measured at 25 °C, consistent with the feedstock becoming more viscous at the lower temperature. By contrast, after freezing and re-melting, the data showed *G*’ > *G*” across the entire frequency range, consistent with the specimen having gelled. Moreover, the data at 25 °C appeared to lie above that at 5 °C, which may have been due to the phase change progressing further between the two sets of measurements.

## 3. Discussion

Whilst it remains inside the animal, silk feedstock appears to be essentially stable, for days or even weeks under suitable storage conditions [61]. On the other hand, the results presented here demonstrated that gelation of the native silk feedstock can be initiated in several ways, including shear flow generating sufficient stress, high temperature or freezing. Moreover, following initiation, it appears that the phase change can progress for a considerable time, without requiring continued application of the stimulus, implying a multi-stage process.

In order for native silk feedstock to avoid spontaneous coagulation, it must be either thermodynamically stable or kinetically inert. The former suggests that the liquid feedstock would be energetically favourable over other related phases (such as coagulated fibroin in water); in which case, silk fibres would spontaneously dissolve in water. Instead, we suggest that it is more accurate to regard the native feedstock as being inert. The question then becomes: what must occur to raise the feedstock out of this inertia? Clearly, this stimulus must be relatively subtle; it can be enacted by silkworms and many other arthropods during natural silk spinning, while the results presented here demonstrate that a brief period of higher shear stress (above 5–12 kPa), elevated temperatures (ca. 60 °C) or freezing were sufficient.

Explanations of protein gelation are often given in terms of a balance between hydrophilic and hydrophobic amino-acids and intermolecular interactions moderated by the pre-existing tertiary structure of the protein coil [43,75,76]. Yet a considerable body of literature [49,50,51,52,53,54,55,56] suggests that, prior to coagulation, fibroin is molecularly dissolved in native silk feedstock and adopts a random coil configuration undergoing uniform chain motion [54,55,56], which precludes this conventional explanation. Instead, we propose an alternative hypothesis, based on the native structure of the protein being stabilised by a hydration shell, which can be displaced as a consequence of entropy changes. Elements of this have already been suggested by Porter and Vollrath, in terms of the intimate hydrogen bonding between peptide groups of the protein and water [92,93]. As a test, calculations based on this concept gave a close match with enthalpy and heat capacity changes during the denaturing transition of egg lysozyme [93].

### A New Hypothesis for Fibroin Coagulation: Entropy Driven Dehydration

**Figure 8:**
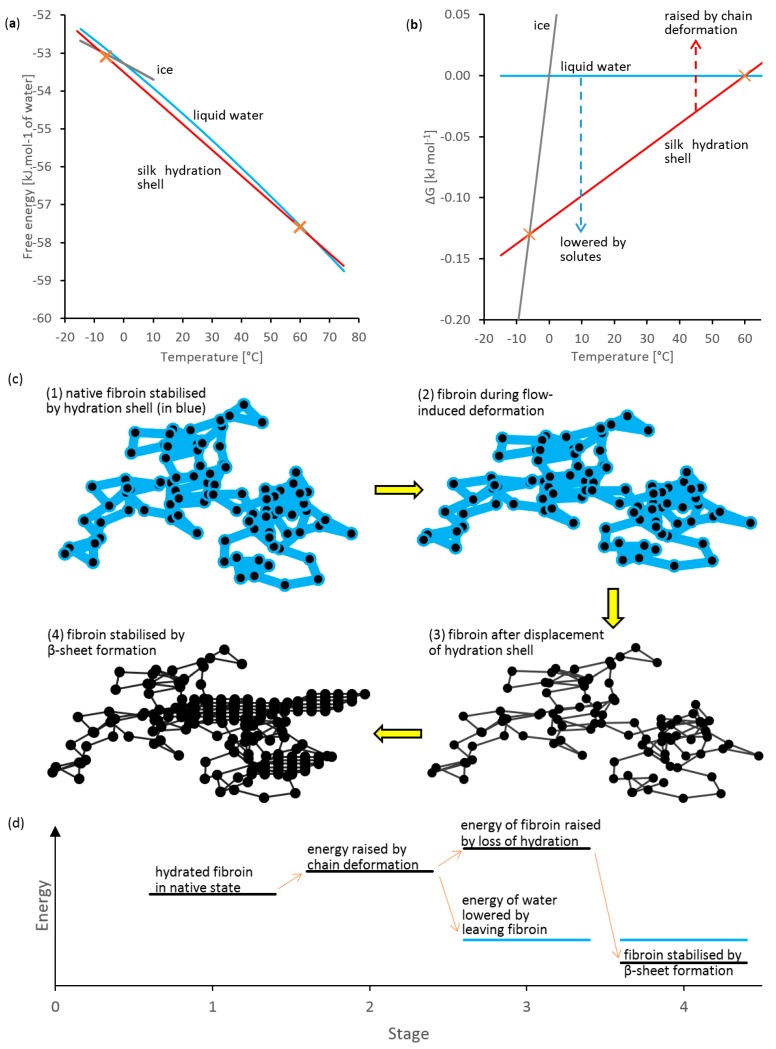
Representative illustration of entropy-driven de-solvation hypothesis: (**a**) free energy vs. temperature plots for ice (grey), water (blue) and fibroin hydration shell (red); (**b**) free energy change from liquid water to ice and hydration shell (assuming linear interpolation); (**c**) representing four stages of flow-induced coagulation (random coil, native hydrated fibroin, flow-induced chain deformation, loss of hydration and β-sheet formation); (**d**) corresponding energy changes.

Perhaps the best starting point for explaining this hypothesis is to consider plots of free energy against temperature for liquid water (in blue) and ice (in grey), as shown in Figure 8a. These were calculated using published thermodynamic data [94,95], as outlined in Appendix A, with the lines for ice and water crossing at 0 °C. As these lines are rather close together and difficult to distinguish, liquid water is used as a reference and the differences in energy (Δ*G*) from this (or its extrapolation to <0 °C) are plotted in Figure 8b. The phase behaviour can then be explained by water seeking the lowest energy state at a given temperature (i.e., ice below 0 °C and liquid water above).

It is proposed that the observed thermal gelation of silk feedstock (at 60 °C) represented the point at which the free energy (or Δ*G*) for the fibroin hydration shell (shown in red) intersected the “water line”. Above this temperature, the fibroin hydration shell would become unstable relative to water and would be lost. Similarly, the gelation on freezing may be explained by crossing the “ice line”. For the present, it is assumed that the free energy (or Δ*G*) plots for the fibroin hydration shell are linear between these points; however, more accurate calculations should be possible using modern modelling methods.

The slope of the fibroin hydrate free energy plot in Figure 8a is slightly less than that of water, corresponding to a decrease in entropy due to its association with the protein. Assuming the enthalpy and entropy to be constant, using:

(4) $$\frac{dG}{dT}=-S$$

gave a value of *S* = 68.2 J·mol^−1^·K^−1^, between −6 and 60 °C, for water within the hydration shell. By comparison, an average value of 70.4 J·mol^−1^·K^−1^ was estimated for liquid water over the same temperature range. Hence, this corresponded to Δ*S* = −2.2 J·mol^−1^·K^−1^ for the change from liquid water to the hydration shell. Under ambient conditions, this (unfavourable) loss of entropy is compensated for by (favourable) enthalpy changes associated with the strength of hydrogen bonding between water and the protein. Assuming that liquid water and the hydration shell are in equilibrium at 60 °C (i.e., Δ*G* = 0 at 333 K):

(5) ΔH=TΔS

Hence, an enthalpy change of Δ*H* = −733 J·mol^−1^ can be estimated for transfer of water from the bulk to the hydration shell. It is now possible to estimate the stability of the hydration shell; for example: at 25 °C, a value of −77.4 J·mol^−1^ of water is obtained, indicating that the hydration shell is stable relative to bulk water, preventing spontaneous gelation. The entropy loss becomes more important at higher temperatures, however, eventually dominating the enthalpy. Consequently, as its free energy rises above that of liquid water, the hydration shell becomes unstable and is lost from the protein. This allows the formation of new protein-to-protein hydrogen bonds, and the fibroin adopts a β-sheet configuration.

In a similar way, it is also possible to explain how silk gelation occurs in salt solutions. In this case, the addition of solutes reduces the free energy of water to below the silk hydrate; the hydration shell becomes unstable relative to the solution and is lost, allowing protein-to-protein interactions. 

It should be emphasised that this explanation is rather different from the conventional description of a hydrophobic solute, which frustrates the hydrogen bonding requirements of the surrounding water. In that case, the water forms a clathrate (or cage) around the solute, so as to minimise any loss of hydrogen bonding [96]. This restructuring is entropically unfavourable, which results in a free energy penalty for dissolution of the hydrophobic solute in water. To put this in context: Δ*G* of 14.5 and 24.5 kJ·mol^−1^ have been estimated for methane and n-butane respectively, with loss of entropy being the major contribution [96].

By contrast, the present hypothesis involves water forming strong hydrogen bonds to peptide groups within the relatively immobile protein. This reduces the movement (i.e., the translational, rotational and vibrational modes) of water, thereby decreasing entropy and raising its free energy.

The flow-induced phase change can now be explained within this proposal. In essence, energy must be added to the hydration shell, in order to overcome its favorable free energy relative to water. We suggest that this occurs through changes in the conformation of the hydrated protein, as a result of flow [87,88,89,90,91], which is observed rheologically in the form of stress. In line with rubber elasticity theory [84,88,97,98], it is expected that deformation of the protein coil will be entropic, involving changes in bond angles and restrictions in its conformational flexibility (as indicated in Figure 8c) rather than bond stretching (which would be enthalpic). Hence, we propose that the free energy of the hydration shell will also be raised through a reduction in its entropy, due to association with the stretched polymer coil, as shown in Figure 8b–d.

In a conventional rubber (e.g., with cross-linked hydrocarbon chains) undergoing deformation, the resulting increase in (potential) energy is stored as a decrease in entropy until the constraints are removed; then the rubber returns to its undeformed state, regaining the lost entropy and reducing its energy. We suggest that another option exists for the deformed fibroin hydrate system, however; the raised energy of the hydration shell (with reduced entropy through its association with the polymer) can be recovered by dissociating from the protein, to become “free” water. The loss of hydration probably raises the energy of the protein further, but this can also be recovered by forming strong hydrogen bonds in β-sheets (indicated by stage 4 in Figure 8c).

This hypothesis is consistent with stress, as opposed to shear rate, being the main factor during initiation, as demonstrated in Figure 2 and Figure 5. It can also account for the two-stage process of flow-induced initiation followed by gelation, as observed in this work and elsewhere [49,52,99]. The initiation stage probably involved chain deformation and sufficient loss of hydration to form a physically cross-linked network. This would explain the sudden build-up of residual stress observed under quasi-static conditions. The subsequent increase in gel strength (giving further increases in *G*’ or decreases in δ) may be associated with slower β-sheet formation; recent work by Boulet-Audet et al. [99] provides experimental support for this. Alternatively, cross-links developed during the first stage would also produce a decrease in entropy and further initiation. This feedback mechanism could be particularly important as different amino-acid sequences within the fibroin are likely to exhibit different hydrophilicities, producing variations in their ease of dehydration.

Clearly, this hypothesis is only conjectural, at present. It is also highly simplified: it takes no account of differences in hydration between different amino-acids, the thickness of the hydration shell, whether the dehydration starts locally at certain key positions or evenly over the entire protein. Consequently, further testing and refinement is required, which will be the subject of subsequent publications.

Finally, it may be interesting to note that flow induced phase changes have been observed in other systems, involving different mechanisms. For example: Xie et al. [100] described the flow-induced gelation of charge-stabilised poly(styrene-*co*-acrylate) suspensions. In spite of the obvious differences between that system (involving colloidal particles with radii of 61, 128 or 288 nm) and (molecularly dissolved) protein in native silk feedstocks, some interesting parallels can be found. Firstly, stabilision due to electrostatic repulsion of the adsorbed ions (i.e., the Derjaguin–Landau–Verwey–Overbeek interaction) around the polymer particles plays the same role as the hydration shell around the protein in the native silk feedstock; Secondly, this remains stable until displaced following sufficient energy uptake during flow—although that was essentially kinetic in the colloidal poly(styrene-*co*-acrylate) system, rather than entropic in our hypothesis; Thirdly, the fractal network observed following the gelation of the colloidal particles exhibited similar dimensionality to that expected for proteins [75,76], albeit with around 100-times larger constituent particles.

## 4. Materials and Methods

All experiments were performed using native silk feedstocks, which were obtained as described previously [61]. In brief, *B. mori* silkworms in the fifth instar were housed at ca. 10 °C to delay pupation. As required, fresh specimens of predominantly fibroin solution were excised from the middle posterior (MP) gland sections, using a dissection microscope and tweezers, under cold, distilled water. Handling stresses were kept to a minimum to avoid premature gelation.

The solids content of the feedstock (predominantly fibroin) was determined gravimetrically, after drying the specimen to constant weight, under vacuum at 60 °C, on a small piece of aluminium foil.

### Rheology

Rheological characterisation of the feedstock specimens and subsequent phase change behaviour were observed using a Bohlin Gemini (Malvern Instruments, Malvern, UK) rheometer, incorporating a Peltier (heating and cooling) stage, with a CP1/10 cone and plate geometry (5.00 mm radius, 1° opening angle and 30 µm truncation). A specimen of sufficient volume to completely fill the geometry was placed on the (fixed) plate at 25 °C, as described previously. The cone was lowered into the specimen at the slowest speed possible (ca. 0.1 mm·s^−1^) and excess material ejected from between the cone and plate was not removed, to minimise flow stresses that could have initiated a phase change. A small amount of distilled water was applied around the specimen and the area was enclosed using a loose-fitting plastic cover, to avoid drying and skin formation.

The experiments were performed in several stages. Firstly, in view of the rather large variations observed previously, it was necessary to characterise the flow behaviour under standard conditions (25 °C) for the specimen chosen. This was achieved using a combination of shear flow at a constant rate ($\dot{\gamma }$ = 1 s^−1^) and oscillatory measurements over a frequency range (*f* = 25 to 0.1 Hz), as described previously.

Following the standard characterisation of the specimen, solid-to-liquid phase changes were initiated in situ and observed by further rheological measurements. Flow-induced phase changes were investigated using combinations of brief periods of duration *t*_A_ at higher shear rates ${\dot{\gamma }}_{A}$, followed by further oscillatory measurements or quasi-static stress relaxation measurements (at $\dot{\gamma }$ = 0.0005 s^−1^). In order to accommodate the rapidly changing rheology during gelation, subsequent oscillatory measurements were limited to a single frequency (at 0.1 or 0.2 Hz) or a rapid scan consisting of a few (generally five) points across a shorter frequency range (12.5 to 0.2 Hz). Phase changes due to heating or freezing were induced by changing the specimen temperature using the Peltier stage and observed by oscillatory measurements. More details of these experiments are given in the Results section.

## 5. Conclusions

This work has demonstrated that the gelation of *B. mori* native silk feedstocks can be initiated merely by exceeding a threshold stress during shear flow at a sufficiently high rate. Shear rate per se was less critical for initiation and showed considerable inverse dependence on the viscosity of the feedstock specimens. Other changes (of salts, pH etc.,) were not required, although their possible role in the natural spinning process is not excluded. Following initiation, the gelation can proceed over a considerable time, without the need for continued application of the original stimulus. This suggests that initiation and gelation should be regarded as a two-stage process. The phase change can also be brought about by heating above 60 °C or by freezing (below −6 °C).

In view of the random coil configuration indicated by the literature for fibroin, the conventional explanation of protein gelation appears unsuitable for this material. Instead, a new hypothesis has been suggested, based on entropically driven dehydration, which is able to provide an explanation for the shear-induced phase change, as well as temperature-, freezing- and salt-induced gelation of fibroin.

## Appendix A. Calculation of Free Energy Curves for Ice and Water

Values for the entropy (70.06 J·mol^−1^·K^−1^) and free energy (−54.951 kJ·mol^−1^) of water at 25 °C were obtained by interpolation from the data published by Pistorius and Sharp [94]. Changes due to temperature were then calculated based on heat capacity (*c*_p_) data [95], using standard thermodynamic expressions:

(A1) $$dS=c_{p}(T).\text{ln}\left(\frac{T_{2}}{T_{1}}\right)$$

(A2) dG=−S(T).dT

The free energy of ice and liquid water should be equal at 0 °C (i.e., at *T*_m_ = 273.15 K). Hence, the change in entropy (Δ*S*_m_) at that temperature was calculated from the latent heat of fusion (Δ*H*_m_ = 6.012 kJ·mol^−1^), using:

(A3) $$\Delta S_{m}=\frac{\Delta H_{m}}{T_{m}}$$

The changes in free energy with temperature for ice were then calculated using Equations (A1) and (A2), with the corresponding values of heat capacity.
